# Exploratory Analysis of Pruritus and Sleep Outcomes After LCR35 Supplementation in Maintenance Hemodialysis: A Randomized, Open-Label Controlled Study

**DOI:** 10.3390/life16081291

**Published:** 2026-08-05

**Authors:** Kuo-Chin Hung, Min-Tser Liao, Cai-Mei Zheng, Shih-Feng Chen, Li-Ting Lu, Chia-Ter Chao, Kuo-Cheng Lu

**Affiliations:** 1 Nephrology Department, New Taipei City Hospital, New Taipei City 241204, Taiwan; corey926@gmail.com (K.-C.H.); gregchen93@gmail.com (S.-F.C.); 2 Department of Nursing, University of Kang Ning, Taipei 114311, Taiwan; lyteen69@gmail.com; 3 Department of Pediatrics, Taoyuan Armed Forces General Hospital Hsinchu Branch, Hsinchu 30054, Taiwan; liaoped804h@yahoo.com.tw; 4 Department of Pediatrics, Tri-Service General Hospital, National Defense Medical University, Taipei 114201, Taiwan; 5 Division of Nephrology, Department of Internal Medicine, Taipei Medical University-Shuang Ho Hospital, New Taipei City 235057, Taiwan; 11044@s.tmu.edu.tw; 6 Division of Nephrology, Department of Internal Medicine, School of Medicine, College of Medicine, Taipei Medical University, Taipei 110052, Taiwan; 7 Taipei Medical University-Research Center of Urology and Kidney (TMU-RCUK), School of Medicine, College of Medicine, Taipei Medical University, Taipei 110052, Taiwan; 8 Institute of Environmental and Occupational Health Sciences, National Taiwan University College of Public Health, Taipei 100025, Taiwan; 9 Division of Nephrology, Department of Internal Medicine, National Taiwan University Hospital and National Taiwan University College of Medicine, Taipei 100225, Taiwan; 10 Graduate Institute of Toxicology, National Taiwan University College of Medicine, Taipei 100225, Taiwan; 11 Graduate Institute of Medical Education and Bioethics, National Taiwan University College of Medicine, Taipei 100225, Taiwan; 12 Division of Nephrology, Department of Medicine, Taipei Tzu Chi Hospital, Buddhist Tzu Chi Medical Foundation, New Taipei City 23142, Taiwan; kuochenglu@gmail.com; 13 Division of Nephrology, Department of Medicine, Fu-Jen Catholic University Hospital, School of Medicine, Fu-Jen Catholic University, New Taipei City 24352, Taiwan

**Keywords:** LCR35, hemodialysis, uremic pruritus, sleep disturbance, symptom burden, 5-D Itch Scale, Pittsburgh Sleep Quality Index

## Abstract

Background/Objectives: Uremic pruritus and sleep disturbance commonly coexist in patients receiving maintenance hemodialysis. This analysis examined whether supplementation with *Lactobacillus casei* var. *rhamnosus* LCR35 was associated with changes in pruritus and sleep outcomes. Methods: This 12-week randomized, open-label, controlled study included 78 patients receiving maintenance hemodialysis who were assigned to LCR35 supplementation (*n* = 38; three sachets daily) or usual care without probiotic supplementation (*n* = 40). No placebo was used. Outcomes were Pittsburgh Sleep Quality Index (PSQI) and 5-D Itch Scale results. We used analysis of covariance (ANCOVA) for between-group analyses, with the week-12 score as the dependent variable and the corresponding baseline score as a covariate. Results: In the LCR35 group, descriptive improvements were observed in the 5-D Itch Scale and selected PSQI outcomes. However, none of the baseline-adjusted between-group differences was statistically significant: for 5-D Itch Scale, −1.48 (95% confidence interval (CI), −3.64 to 0.68; *p* = 0.176); for global PSQI, −0.80 (95% CI, −2.09 to 0.50; *p* = 0.225); for PSQI sleep disturbance, −0.07 (95% CI, −0.27 to 0.13; *p* = 0.482); and use of sleep medications, −0.28 (95% CI, −0.67 to 0.12; *p* = 0.163). In the LCR35 group, the exploratory association between changes in pruritus and the PSQI sleep-disturbances component was significant before, but not after, false-discovery-rate (FDR) correction (*p* = 0.007; FDR *q* = 0.055). Conclusions: Our preliminary findings raised the possibility that LCR35 might modestly influence pruritus and specific sleep disturbances. However, our results do not firmly establish a treatment effect, nor a patient phenotype most likely to benefit.

## 1. Introduction

The symptom burden of chronic kidney disease (CKD) is heterogeneous and frequently under-recognized [1,2,3]. In patients receiving maintenance hemodialysis, pruritus and sleep disturbance are common and often coexist [4,5,6,7,8]. Among these complaints, CKD-associated pruritus is particularly difficult to manage, since it is known to be multifactorial [9]. However, existing treatments for CKD-associated pruritus and sleep disturbance are frequently suboptimal and with variable efficacy. We therefore chose to assess other potential treatment approaches for these complaints, such as probiotic supplementation.

Prior reports suggested that probiotic interventions may influence sleep and uremic symptom burden through changes in gut microbial composition, microbial metabolites, immune signaling, and gut–brain communication in patients with kidney dysfunction. In a separate randomized, open-label study of patients receiving hemodialysis, LCR35, a form of freeze-dried *Lactobacillus casei rhamnosus*, supplementation was associated with improvements in global Pittsburgh Sleep Quality Index (PSQI) scores and sleep duration, alterations in gut microbiota, and lower indoxyl sulfate (IS) concentrations [10]. Placebo-controlled trials in populations without CKD have also reported improved sleep outcomes after probiotic supplementation [11,12]. These findings provide biological and clinical context for using probiotics to manage these CKD complications, but fail to consistently establish efficacy nor the mechanisms of action in the present literature.

To see whether gut microbiota was potentially modulated, we included IS and p-cresol as the exploratory markers of gut-derived metabolites. IS is a protein-bound uremic solute, whereas p-cresol is a microbial phenolic precursor that is predominantly conjugated in vivo to p-cresyl sulfate and p-cresyl glucuronide [13,14]. Results of these biomarkers were therefore considered exploratory biochemical surrogates, or a secondary post hoc analysis of this study, in order to evaluate the potential changes in gut microbiota. They were hypothesis-generating and were not meant for subsequent mediation analysis, such as of the pruritus-defined responder phenotype, nor a confirmatory evaluation of LCR35 efficacy.

## 2. Materials and Methods

### 2.1. Study Design and Participants

This 12-week randomized, open-label, controlled study compared a probiotic, LCR35 supplementation, with usual care in patients receiving maintenance hemodialysis. Participants were recruited from a single center and assigned in a 1:1 ratio to the LCR35 or control group. The intervention group received LCR35 supplementation, whereas the control group continued usual care without probiotic supplementation. No placebo was used, and participants and investigators were aware of treatment allocation.

Eligible participants were adults aged 20–100 years who had received regular hemodialysis for at least three months and had a global PSQI score > 5 at screening, indicating poor sleep quality. Pruritus severity was assessed prospectively at enrollment using the 5-D Itch Scale. However, neither the presence of pruritus nor a minimum 5-D Itch Scale score was required for eligibility in this study, because the original trial was focused on sleep quality rather than pruritus. Exclusion criteria were the inability to complete questionnaires, pregnancy, malignancy within the previous six months, active infection or inflammatory disease, known probiotic allergy, or dairy intolerance.

The study records confirmed 1:1 random assignment but did not contain enough information to reconstruct the sequence-generation method, including whether a computer-generated sequence, blocking, or stratification was used, or to verify allocation concealment. The adequacy of random sequence generation and allocation concealment therefore could not be independently assessed. The trial was not prospectively registered. These reporting limitations were considered in the risk-of-bias assessment.

### 2.2. Intervention

Participants in the intervention group received Antibiophilus Powder, a single-strain probiotic preparation containing a freeze-dried culture of *Lactobacillus casei var. rhamnosus*, designated LCR35 in the study protocol. According to the product label, each sachet contained at least 1.5 × 10^8^ colony-forming units (CFUs). Participants were instructed to take three sachets daily for 12 weeks, corresponding to a minimum total daily dose of 4.5 × 10^8^ CFU, at consistent times each day. The listed excipients were potato starch, lactose, and dried milk extract. No specific dietary or lifestyle changes were required, and the control group continued usual hemodialysis care without probiotic supplementation. Because the study did not include a formal quantitative adherence assessment or adherence-rate calculation, the adherence percentage was unavailable.

### 2.3. Sample Size

The prespecified primary outcome of the original randomized study was the change in global PSQI score after 12 weeks. No formal a priori sample-size calculation was performed; therefore, the expected effect size, statistical power, and significance level were not specified for enrollment planning. The final sample comprised 78 randomized participants. A retrospective design-sensitivity analysis showed that group sizes of 38 and 40 provided 80% power, at a two-sided α level of 0.05, to detect a standardized between-group effect of approximately 0.64. This analysis was conducted only to characterize the statistical sensitivity of the available sample and was not used to justify the original enrollment target. No separate sample-size or power calculation was performed for the secondary, post hoc analysis.

### 2.4. Outcome Measures

The 5-D Itch Scale was administered prospectively to assess pruritus severity. This brief multidimensional instrument evaluates itch degree, duration, direction, disability, and distribution and has been validated for chronic pruritus across ethnic groups [15,16]. The present pruritus-centered framing, including the designation of change in the 5-D Itch Scale as the principal symptom outcome, was not part of the originally prespecified primary analysis and is therefore secondary, post hoc, and hypothesis-generating. Global PSQI and its component scores were evaluated as sleep-related outcomes. Associations between changes in pruritus and changes in PSQI components were examined in exploratory post hoc analyses and were not interpreted as mediation analyses. Serum IS and p-cresol were measured as exploratory surrogates of gut-derived metabolites; the p-cresol assay quantified p-cresol rather than p-cresyl sulfate. Assessments were performed at baseline and at the end of the 12-week intervention.

### 2.5. Laboratory Analysis

Serum IS was measured using the Human IS (serum indoxyl sulfate) enzyme-linked immunosorbent assay (ELISA) kit (catalog no. ELK9248), and p-cresol was measured using the Human p-cresol ELISA Kit (catalog no. ELK10889; ELK [Wuhan] Biotechnology Co., Ltd., Wuhan, China). Both assays were sandwich enzyme immunoassays. According to the manufacturer’s protocol, 100 μL of standard or serum sample was added to antibody-precoated wells and incubated for 80 min at 37 °C. After three washes, 100 μL of biotinylated detection antibody was added and incubated for 50 min at 37 °C. After three additional washes, 100 μL of streptavidin-horseradish peroxidase (HRP) was added and incubated for 50 min at 37 °C. The plates were then washed five times, developed with 90 μL of TMB substrate for 20 min at 37 °C in the dark, treated with 50 μL of stop solution, and read at 450 ± 10 nm. Concentrations were calculated from blank-corrected standard curves. The manufacturer reports a sensitivity of 12.1 ng/mL and an analytical range of 31.25–2000 ng/mL for IS, and a sensitivity of 4.8 pg/mL and an analytical range of 15.63–1000 pg/mL for p-cresol. For both kits, the reported intra-assay and inter-assay coefficients of variation are <8% and <10%, respectively. Historical plate-level quality-control records, participant-level dilution details, and information on whether the IS result represented the free or total fraction were not retained. No prespecified rule for excluding statistical outliers was documented. The biomarker findings were interpreted as exploratory in nature and not used to support the mechanism of action.

### 2.6. Statistical Analysis

Continuous variables are summarized as mean ± standard deviation (SD), and categorical variables as counts and percentages. Paired Student t-tests were used for baseline-to-week-12 comparisons within each group; these analyses were considered descriptive and secondary. The principal between-group analyses used analysis of covariance (ANCOVA), with the week-12 score as the dependent variable, treatment group as the fixed factor, and the corresponding baseline score as a covariate. We report adjusted marginal means, adjusted between-group mean differences with 95% confidence intervals (CIs), two-sided *p* values, and covariate-adjusted Hedges g values with 95% CIs. For symptom scores, negative adjusted differences favor the presence of LCR35 effects. Homogeneity of regression slopes was assessed by including a treatment-group-by-baseline interaction; no evidence of interaction was found for the four principal outcomes (all *p* ≥ 0.154). Because the symptom-cluster analysis was post hoc and exploratory, no multiplicity adjustment was applied across the four principal ANCOVA comparisons. Estimates were interpreted primarily according to their magnitude and CIs. A two-sided *p* value < 0.05 was considered statistically significant.

**Figure 1:**
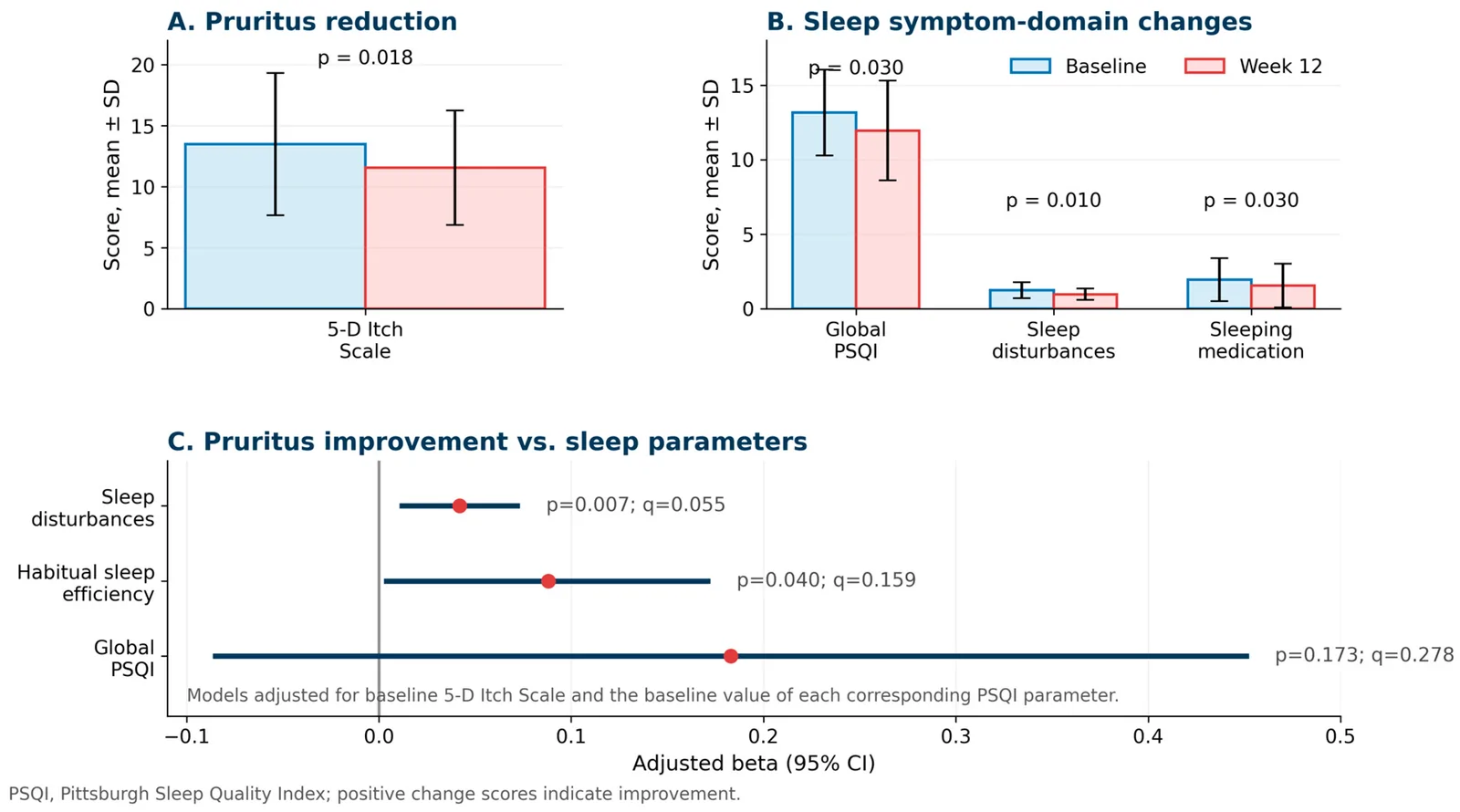
Descriptive within-group changes and exploratory associations between pruritus and sleep. (**A**) Change in the 5-D Itch Scale score from baseline to week 12 within the LCR35 group. (**B**) Changes in global PSQI, the PSQI sleep-disturbance component, and use of sleeping medication from baseline to week 12 within the LCR35 group. Panels A and B depict descriptive within-group comparisons and should not be interpreted as between-group treatment effects; baseline-adjusted between-group estimates are reported in Table 1. (**C**) Exploratory FDR-adjusted associations between improvement in pruritus and changes in selected sleep measures. Positive change scores indicate improvement. PSQI, Pittsburgh Sleep Quality Index; CI, confidence interval; FDR, false discovery rate.

For the exploratory pruritus-sleep analysis, change scores were calculated as baseline minus week-12 values, so positive values indicated improvement. Analyses were limited to participants who received LCR35 (*n* = 38). Spearman correlations were calculated between changes in the 5-D Itch Scale and changes in global PSQI and individual PSQI component scores. Separate linear regression models were then fitted for each PSQI change score, with change in the 5-D Itch Scale as the independent variable and adjustment for baseline 5-D Itch Scale score and the baseline value of the corresponding PSQI measure. The Benjamini–Hochberg method was used to control the false discovery rate (FDR) across the PSQI measures. These analyses were explicitly considered exploratory and post hoc. Analyses were performed in SPSS and verified using Python-based statistical procedures. Figures were generated using Python with Matplotlib version 3.10.8. (Figure 1) and Microsoft PowerPoint (Supplementary Figure S1).

**Table 1:** Baseline-adjusted between-group analyses of the principal symptom outcomes.

Outcome	Adjusted LCR35 Mean	Adjusted Control Mean	Adjusted Difference (95% CI)	*p* Value	Adjusted Hedges g (95% CI)
5-D Itch Scale	10.66	12.14	−1.48 (−3.64 to 0.68)	0.176	−0.33 (−0.81 to 0.15)
Global PSQI	11.72	12.52	−0.80 (−2.09 to 0.50)	0.225	−0.28 (−0.73 to 0.17)
Sleep disturbances	1.03	1.10	−0.07 (−0.27 to 0.13)	0.482	−0.17 (−0.63 to 0.30)
Use of sleeping medication	1.58	1.85	−0.28 (−0.67 to 0.12)	0.163	−0.32 (−0.76 to 0.13)

Adjusted means were estimated at the overall mean of the corresponding baseline outcome. The adjusted difference was calculated as LCR35 minus control; negative values favor LCR35 for symptom scores. Adjusted Hedges g was calculated by standardizing the ANCOVA group coefficient using the model residual SD and applying a small-sample correction. CI, confidence interval; PSQI, Pittsburgh Sleep Quality Index.

### 2.7. Ethics Approval and Informed Consent

The study was conducted in accordance with the Declaration of Helsinki and approved by the Institutional Review Board of Min-Sheng General Hospital (approval no. MSIRB2023003; 11 April 2023). All participants provided written informed consent before enrollment and before any study-specific procedure.

## 3. Results

### 3.1. Baseline Characteristics

The analysis included 78 patients receiving maintenance hemodialysis: 38 in the LCR35 group and 40 in the control group. Table 2 summarizes the baseline demographic and clinical characteristics. No statistically significant between-group differences were observed in age, sex, dialysis vintage, diabetes mellitus, hemoglobin, calcium, phosphate, intact parathyroid hormone, or most routine biochemical measures. Baseline alanine aminotransferase (ALT) was higher in the LCR35 group than in the control group (18.58 ± 14.63 vs. 12.95 ± 5.55 IU/L; *p* = 0.031), as was total iron-binding capacity (TIBC; 210.37 ± 37.20 vs. 184.95 ± 32.17 μg/dL; *p* = 0.002). Aspartate aminotransferase, alkaline phosphatase, and albumin did not differ significantly. These baseline imbalances were not interpreted as causally related to the symptom outcomes.

### 3.2. Pruritus Outcome

Because baseline pruritus severity was higher in the LCR35 group, the principal inference was based on ANCOVA. The baseline-adjusted estimated mean 5-D Itch Scale scores at week 12 were 10.66 in the LCR35 group and 12.14 in the control group. The adjusted between-group difference was −1.48 points (95% CI, −3.64 to 0.68; *p* = 0.176), with an adjusted Hedges g of −0.33 (95% CI, −0.81 to 0.15). Thus, the adjusted between-group difference was not statistically significant (Table 1). In descriptive within-group analyses, the 5-D Itch Scale score decreased from 13.50 ± 5.84 at baseline to 11.55 ± 4.69 at week 12 in the LCR35 group (*p* = 0.018) and changed from 9.60 ± 4.58 to 11.30 ± 5.30 in the control group (*p* = 0.067) (Table 3). Figure 1A displays these descriptive within-group changes.

### 3.3. Sleep Outcomes and PSQI Components

In the baseline-adjusted ANCOVA, none of the principal sleep outcomes differed significantly between groups. The adjusted between-group difference was −0.80 points for global PSQI (95% CI, −2.09 to 0.50; *p* = 0.225; adjusted Hedges g, −0.28 [95% CI, −0.73 to 0.17]), −0.07 points for sleep disturbances (95% CI, −0.27 to 0.13; *p* = 0.482; adjusted Hedges g, −0.17 [95% CI, −0.63 to 0.30]), and −0.28 points for use of sleeping medication (95% CI, −0.67 to 0.12; *p* = 0.163; adjusted Hedges g, −0.32 [95% CI, −0.76 to 0.13]) (Table 1). In descriptive within-group analyses, global PSQI decreased from 13.16 ± 2.87 at baseline to 11.95 ± 3.34 at week 12 in the LCR35 group (*p* = 0.030). The sleep-disturbance and use-of-sleeping-medication components also decreased within the LCR35 group (Table 4). Figure 1B displays these descriptive changes.

### 3.4. Exploratory Biomarker Outcomes

The biomarker analyses were descriptive and exploratory. In the LCR35 group, serum IS decreased from 231.97 ± 334.15 ng/mL at baseline to 113.61 ± 87.95 ng/mL at week 12 (within-group *p* = 0.014). The control group also showed a numerical decrease, but the within-group change was not statistically significant. In the LCR35 group, p-cresol decreased from 20.01 ± 12.92 pg/mL to 14.81 ± 11.57 pg/mL (within-group *p* = 0.075); the within-group change in the control group was also not statistically significant (Table 5). These comparisons were not designated as principal treatment-effect analyses and do not establish efficacy or a mechanism of action.

### 3.5. Exploratory Association Between Changes in Pruritus and Sleep Parameters in the LCR35 Group

The exploratory association analyses were limited to participants who received LCR35 (*n* = 38). In the unadjusted Spearman analyses, change in the 5-D Itch Scale was not significantly associated with change in global PSQI. The strongest unadjusted association involved use of sleeping medication, but its direction was inverse and it did not persist after adjustment. In the adjusted regression models, which controlled for baseline pruritus severity and the baseline value of the corresponding PSQI measure, improvement in pruritus was associated most strongly with improvement in the PSQI sleep-disturbance component (adjusted β = 0.042; 95% CI, 0.012 to 0.072; *p* = 0.007). However, this association did not meet the FDR-adjusted significance threshold (*q* = 0.055). Habitual sleep efficiency also showed a nominal association before correction for multiple comparisons (Table 6). These results are hypothesis-generating rather than confirmatory. Figure 1C displays the principal adjusted associations.

### 3.6. Baseline-Adjusted Between-Group Comparisons

In summary, all four baseline-adjusted estimates favored LCR35 numerically, but none reached statistical significance (Table 1). The adjusted difference was −1.48 points for the 5-D Itch Scale (95% CI, −3.64 to 0.68; *p* = 0.176), −0.80 points for global PSQI (95% CI, −2.09 to 0.50; *p* = 0.225), −0.07 points for the PSQI sleep-disturbances component (95% CI, −0.27 to 0.13; *p* = 0.482), and −0.28 points for use of sleeping medication (95% CI, −0.67 to 0.12; *p* = 0.163). The CIs were compatible with both a modest benefit and no effect. These analyses therefore do not provide confirmatory evidence of efficacy.

### 3.7. Safety, Treatment Adherence, and Concomitant Medications

The study records contained no reports of adverse events, infection-related events, gastrointestinal symptoms, or antibiotic use during follow-up. The original study did not include a formal quantitative adherence measure, such as returned-sachet counts, participant diaries, or electronic monitoring, and no adherence threshold was predefined. The proportion of prescribed sachets consumed and the proportion of participants meeting an adherence criterion therefore could not be determined. No changes in antihistamine, hypnotic or sedative, or emollient use, or in dialysis adequacy, were reported during follow-up.

## 4. Discussion

The current analysis of a randomized, open-label, controlled study found descriptive within-group improvements in pruritus and selected sleep outcomes after LCR35 supplementation. However, after adjustment for the corresponding baseline values, the between-group differences in the 5-D Itch Scale, global PSQI, PSQI sleep disturbances, and use of sleeping medication were not statistically significant. Although the adjusted estimates generally favored LCR35, all CIs included no effect. The analysis therefore did not establish that LCR35 improved pruritus or sleep, or that any change in sleep was caused or mediated by improvement in pruritus.

The within-group decrease in the 5-D Itch Scale is clinically relevant because CKD-associated pruritus remains difficult to treat [9]. However, baseline pruritus severity was higher in the LCR35 group, and the baseline-adjusted between-group difference was not statistically significant (adjusted difference, −1.48 points; 95% CI, −3.64 to 0.68; adjusted Hedges g, −0.33 [95% CI, −0.81 to 0.15]). Conventional approaches, including topical therapies, gabapentinoids, opioid-receptor modulators, dialysis optimization, and toxin-directed strategies, may be inadequate or poorly tolerated in some patients [9,17]. Probiotic supplementation remains a low-burden strategy that merits further evaluation, but any symptomatic benefit must be confirmed in an adequately powered, placebo-controlled trial. Trials of difelikefalin and related analyses demonstrate the broader clinical importance of reducing itch-related sleep disruption and impairment in quality of life [18,19,20].

The descriptive sleep findings were limited to selected domains: global PSQI, sleep disturbances, and use of sleeping medication decreased within the LCR35 group. However, the corresponding baseline-adjusted between-group differences were not statistically significant, and the standardized effect estimates were small, with CIs suggesting an absence of effect. The present data therefore do not establish that LCR35 improves sleep. Indeed, sleep quality in hemodialysis is multifactorial and may be influenced by comorbidities, dialysis-related discomfort, psychological factors, medications, expectation effects, pruritus, and other symptoms.

Probiotics may also affect sleep through pathways independent of pruritus. In a separate open-label hemodialysis patient study, two daily sachets of LCR35 for 12 weeks were associated with improvements in global PSQI score and sleep duration, changes in the gut microbiota, and lower IS concentrations, whereas the 5-D Itch Scale did not change significantly [10]. This pattern is compatible with a gut-microbiota–sleep pathway that does not require measurable improvement in pruritus. In populations without CKD, a 12-week placebo-controlled trial reported improved sleep efficiency together with changes in the microbiota and microbial metabolites [11], and a 28-day double-blind, placebo-controlled trial reported improvements in PSQI, sleep efficiency, and sleep latency after a multispecies probiotic intervention [12]. Because these studies used different strains or mixtures, doses, populations, and sleep assessments, they cannot confirm an LCR35 effect in hemodialysis. Nevertheless, they broaden the mechanistic context beyond a pruritus-centered explanation. The present study did not include stool sequencing, metabolomics, inflammatory profiling, or objective sleep monitoring and therefore cannot determine whether microbiota-related pathways contributed to the observed within-group changes.

Our findings should also be considered in relation to the meta-analyses cited in the Introduction. Rehman et al. pooled 28 studies and estimated that approximately 40% of hemodialysis patients had disturbed sleep and that approximately 50% had sleep disturbance attributed to CKD-associated pruritus, although heterogeneity was substantial [5]. Koh et al. similarly reported bidirectional associations between CKD and several sleep disorders [21]. These pooled findings support the direction and clinical relevance of the pruritus–sleep relationship, but they summarize prevalence or observational associations rather than probiotic treatment effects and therefore are not directly comparable with our ANCOVA estimates. In the present analysis, the adjusted standardized effects were small (Hedges g: −0.33 for the 5-D Itch Scale and −0.28 for global PSQI), and both CIs included no effect. Differences from stronger associations reported previously may reflect the single-strain formulation and dose, the 12-week intervention, enrollment based on PSQI > 5 rather than clinically significant pruritus, inclusion of participants with minimal baseline itching, the baseline imbalance in pruritus severity, the single-center Asian population, the open-label design without placebo control, and reliance on patient-reported outcomes.

The within-LCR35 association analysis requires particular caution. Improvement in the 5-D Itch Scale was nominally associated with improvement in the PSQI sleep-disturbance component after adjustment for both baseline scores, but the association did not meet the FDR-adjusted significance threshold (*q* = 0.055). The model included only 38 participants from the intervention group, did not compare the association between randomized groups, and was not a formal mediation analysis. It therefore generated a hypothesis for future testing but did not show that LCR35 improved pruritus-driven sleep disturbance or that patients with nocturnal itching are more likely to benefit.

Microbiome-related mechanisms relevant to pruritus may extend beyond the intestinal compartment. Changes in the skin microbiome and microbial fermentation products have also been implicated in CKD-associated pruritus [22,23]. However, this study assessed neither the gut nor the skin microbiota, and therefore cannot establish that LCR35 influenced pruritus through a microbiome-dependent pathway.

IS and p-cresol were measured to provide exploratory biochemical context for a probiotic intervention. IS is a gut-derived, protein-bound uremic solute. p-cresol is generated by microbial fermentation and is predominantly converted in vivo to p-cresyl sulfate and p-cresyl glucuronide [13,14]. The result obtained with the ELK10889 p-cresol assay is therefore not equivalent to a p-cresyl sulfate concentration. Although trials of probiotics, prebiotics, or synbiotics in dialysis populations have evaluated IS and p-cresyl sulfate [24], the p-cresol measurements in this study are not directly comparable with that literature. Evidence linking protein-bound uremic toxins to pruritus is inconsistent across observational studies [25,26]. The within-group decrease in IS and the nonsignificant trend in p-cresol were descriptive, exploratory findings and were not prespecified as principal treatment-effect analyses. They do not establish that changes in either biomarker caused changes in pruritus or sleep.

The baseline differences in ALT and TIBC also require some attention. ALT was higher in the LCR35 group, although mean values were low in absolute terms; aminotransferase activities are often reduced in patients receiving hemodialysis because of factors such as hemodilution and pyridoxine deficiency [27]. Pruritus of hepatobiliary origin is usually evaluated in the context of a cholestatic biochemical pattern, particularly a disproportionate increase in alkaline phosphatase, together with gamma-glutamyl transferase and bilirubin, rather than an isolated difference in ALT. Because alkaline phosphatase and AST did not differ significantly between groups, the available data do not indicate an obvious cholestatic explanation for the baseline difference in pruritus severity. However, gamma-glutamyl transferase and bilirubin were unavailable, preventing a complete hepatobiliary function assessment. The higher TIBC may reflect differences in iron or nutritional status, but it cannot be interpreted as causally related to pruritus or sleep. Neither ALT nor TIBC was prespecified as a symptom-related covariate, and residual confounding cannot be excluded.

Strengths of this study include the prospective collection of validated patient-reported symptom measures and the inclusion of both symptom and exploratory biomarker outcomes. Several limitations, however, substantially restrict interpretation. First, the study was open-label and lacked a placebo control. Because pruritus and sleep were patient-reported, expectation bias, reporting bias, and nonspecific intervention effects may have contributed to the within-group changes. Second, although the retained records confirmed 1:1 random assignment, they did not allow verification of sequence generation or allocation concealment, and the trial was not prospectively registered. These reporting limitations increase uncertainty about risk of bias and reproducibility. Third, the original study did not include a formal a priori sample-size calculation. The available sample was sufficiently powered only for moderate-to-large standardized effects, whereas the observed adjusted effects were small and imprecise. In addition, no formal quantitative adherence measure was collected.

Baseline pruritus severity was higher in the LCR35 group, making baseline-adjusted between-group analysis essential and limiting the within-group paired comparisons to descriptive interpretation. The exploratory pruritus-sleep association analysis included only 38 LCR35-treated participants and did not meet the FDR-adjusted significance threshold. Because a separate pre-randomization screening log was unavailable, the numbers assessed for eligibility and excluded, together with the reasons for exclusion, could not be reliably reconstructed. For the biomarker analyses, the ELK kit identities and principal assay procedures were confirmed; however, historical plate-level quality-control records, participant-level dilution details, information on whether IS represented the free or total fraction, and a prespecified rule for handling outliers were unavailable. Moreover, p-cresol rather than p-cresyl sulfate was measured, and these analytes are not interchangeable. The biomarker findings therefore require cautious, nonmechanistic interpretation. The observed decrease in use of sleeping medication may also have reflected clinical medication-management decisions, although the study records documented no changes in relevant concomitant medications. Future studies should use a double-blind, placebo-controlled design; register the protocol prospectively; document sequence generation and allocation concealment; enroll or stratify participants according to a prespecified, clinically relevant threshold for pruritus; incorporate formal adherence monitoring and objective sleep assessment; and include an adequately powered sample.

## 5. Conclusions

In this analysis of a randomized, open-label study, pruritus and selected sleep outcomes improved descriptively within the LCR35 group, but the baseline-adjusted between-group differences were not statistically significant. The within-LCR35 association between changes in pruritus and the PSQI sleep-disturbances component did not meet the FDR-adjusted significance threshold and did not establish an effect of pruritus improvement on sleep. The biomarker findings were exploratory and did not establish a mechanism of action. Accordingly, these data should not be interpreted as evidence that LCR35 is efficacious. Larger, adequately powered, prospectively registered, double-blind, placebo-controlled trials are needed, with prespecified symptom outcomes, documented allocation procedures, objective adherence monitoring, objective sleep assessment, and microbiome or metabolomic measurements.

## Figures and Tables

**Table 2:** Baseline demographic and clinical characteristics of the LCR35 and control groups.

Variable	LCR35 Group (*n* = 38)	Control Group (*n* = 40)	*p* Value
Age (years)	60.0 ± 11.5	64.3 ± 9.8	0.076
Male, *n* (%)	25 (65.8%)	22 (55.0%)	0.478
HD duration (years)	5.85 ± 5.82	6.69 ± 5.79	0.529
Diabetes mellitus, *n* (%)	28 (73.7%)	25 (62.5%)	0.930
HbA1c (%)	6.49 ± 1.38	6.07 ± 1.13	0.258
iPTH (pg/mL)	422.01 ± 353.91	440.13 ± 515.20	0.856
Alkaline phosphatase (IU/L)	77.97 ± 29.92	93.23 ± 82.11	0.277
Calcium (mg/dL)	9.19 ± 1.04	8.93 ± 0.89	0.232
Phosphate (mg/dL)	4.98 ± 1.69	5.38 ± 1.45	0.264
Total protein (g/dL)	8.41 ± 10.78	6.48 ± 0.46	0.276
Albumin (g/dL)	3.76 ± 0.36	3.67 ± 0.27	0.202
AST (IU/L)	18.87 ± 11.45	15.70 ± 5.09	0.124
ALT (IU/L)	18.58 ± 14.63	12.95 ± 5.55	0.031
Hematocrit (%)	28.89 ± 4.52	28.56 ± 4.33	0.742
Hemoglobin (g/dL)	9.75 ± 1.48	9.72 ± 1.50	0.930
Uric acid (mg/dL)	6.73 ± 1.51	7.84 ± 1.46	0.639
Triglyceride (mg/dL)	137.32 ± 116.48	141.45 ± 89.27	0.861
Total cholesterol (mg/dL)	147.82 ± 42.62	137.05 ± 33.40	0.220
Ferritin (ng/mL)	612.70 ± 371.35	691.57 ± 465.40	0.410
TIBC (µg/dL)	210.37 ± 37.20	184.95 ± 32.17	0.002

Data are presented as mean ± SD unless otherwise indicated. HD, hemodialysis; iPTH, intact parathyroid hormone; AST, aspartate aminotransferase; ALT, alanine aminotransferase; TIBC, total iron-binding capacity.

**Table 3:** Descriptive within-group changes in pruritus severity measured using the 5-D Itch Scale.

Outcome	LCR35 Baseline	LCR35 Week 12	*p* Value	Control Baseline	Control Week 12	*p* Value
5-D Itch Scale score	13.50 ± 5.84	11.55 ± 4.69	0.018	9.60 ± 4.58	11.30 ± 5.30	0.067

**Table 4:** Descriptive changes in PSQI scores within the LCR35 group.

PSQI Parameter	Baseline	Week 12	*p* Value
Global PSQI score	13.16 ± 2.87	11.95 ± 3.34	0.030
Subjective sleep quality	1.97 ± 0.82	1.84 ± 0.79	0.440
Sleep latency	2.50 ± 0.92	2.21 ± 1.19	0.170
Sleep duration	2.45 ± 0.72	2.55 ± 0.72	0.500
Habitual sleep efficiency	2.45 ± 0.80	2.26 ± 0.98	0.320
Sleep disturbances	1.24 ± 0.54	0.97 ± 0.37	0.010
Use of sleeping medication	1.95 ± 1.43	1.55 ± 1.46	0.030
Daytime dysfunction	0.61 ± 0.92	0.55 ± 0.83	0.770

**Table 5:** Exploratory serum IS and p-cresol concentrations before and after the intervention in the LCR35 and control groups.

Biomarker	LCR35 Baseline	LCR35 Week 12	*p* Value	Control Baseline	Control Week 12	*p* Value
Indoxyl sulfate (ng/mL)	231.97 ± 334.15	113.61 ± 87.95	0.014	195.12 ± 230.82	166.04 ± 182.47	0.522
p-cresol (pg/mL)	20.01 ± 12.92	14.81 ± 11.57	0.075	25.72 ± 25.66	20.96 ± 18.17	0.535

Data are presented as mean ± SD.

**Table 6:** Exploratory associations between changes in pruritus and sleep parameters within the LCR35 group.

Sleep Parameter	*n*	Spearman Rho	*p* Value	Adjusted β	95% CI	Adjusted *p*	FDR *q*
Global PSQI score	38	0.197	0.236	0.183	−0.085 to 0.451	0.173	0.278
Subjective sleep quality	38	0.125	0.453	0.056	−0.013 to 0.125	0.110	0.221
Sleep latency	38	0.165	0.321	0.014	−0.091 to 0.120	0.784	0.784
Sleep duration	38	0.147	0.377	0.058	−0.006 to 0.121	0.073	0.196
Habitual sleep efficiency	38	0.199	0.230	0.088	0.004 to 0.171	0.040	0.159
Sleep disturbances	38	0.268	0.104	0.042	0.012 to 0.072	0.007	0.055
Use of sleeping medication	38	−0.324	0.047	−0.026	−0.110 to 0.057	0.527	0.602
Daytime dysfunction	38	0.205	0.217	−0.028	−0.102 to 0.046	0.450	0.600

Change scores were calculated as baseline minus week-12 values; positive values indicate improvement. In each adjusted linear regression model, change in the relevant PSQI measure was the dependent variable and change in the 5-D Itch Scale was the independent variable. Models were adjusted for baseline 5-D Itch Scale score and the baseline value of the corresponding PSQI measure. FDR correction used the Benjamini–Hochberg method.

## Data Availability

The data supporting the findings of this study are available from the first and the corresponding author upon reasonable request.

## Supplementary Materials

The following supporting information can be downloaded at: https://www.mdpi.com/article/10.3390/life16081291/s1, Figure S1: CONSORT 2025 flow diagram showing participant allocation, follow-up, and analysis.

## Abbreviations

**ANOCA**	analysis of covariance
**CKD**	chronic kidney disease
**HD**	hemodialysis
**LCR35**	*Lactobacillus casei* variety *rhamnosus* (study designation LCR35)
**PSQI**	Pittsburgh Sleep Quality Index
**IS**	indoxyl sulfate
**SD**	standard deviation
**CI**	confidence interval
**FDR**	false discovery rate
**iPTH**	intact parathyroid hormone
**AST**	aspartate aminotransferase
**ALT**	alanine aminotransferase
**TIBC**	total iron-binding capacity
**HbA1c**	glycated hemoglobin
**ELISA**	enzyme-linked immunosorbent assay
**IRB**	Institutional Review Board
