# Supplementary material for: Exploratory Analysis of Pruritus and Sleep Outcomes After LCR35 Supplementation in Maintenance Hemodialysis: A Randomized, Open-Label Controlled Study
Source: Life (Basel). 2026 Aug 5;16(8):1291. doi: 10.3390/life16081291 (PMC13514339; doi:10.3390/life16081291)
Supplement: Supplementary file 1 [file life-16-01291-s001.zip › life-4412991-supplementary.pdf]

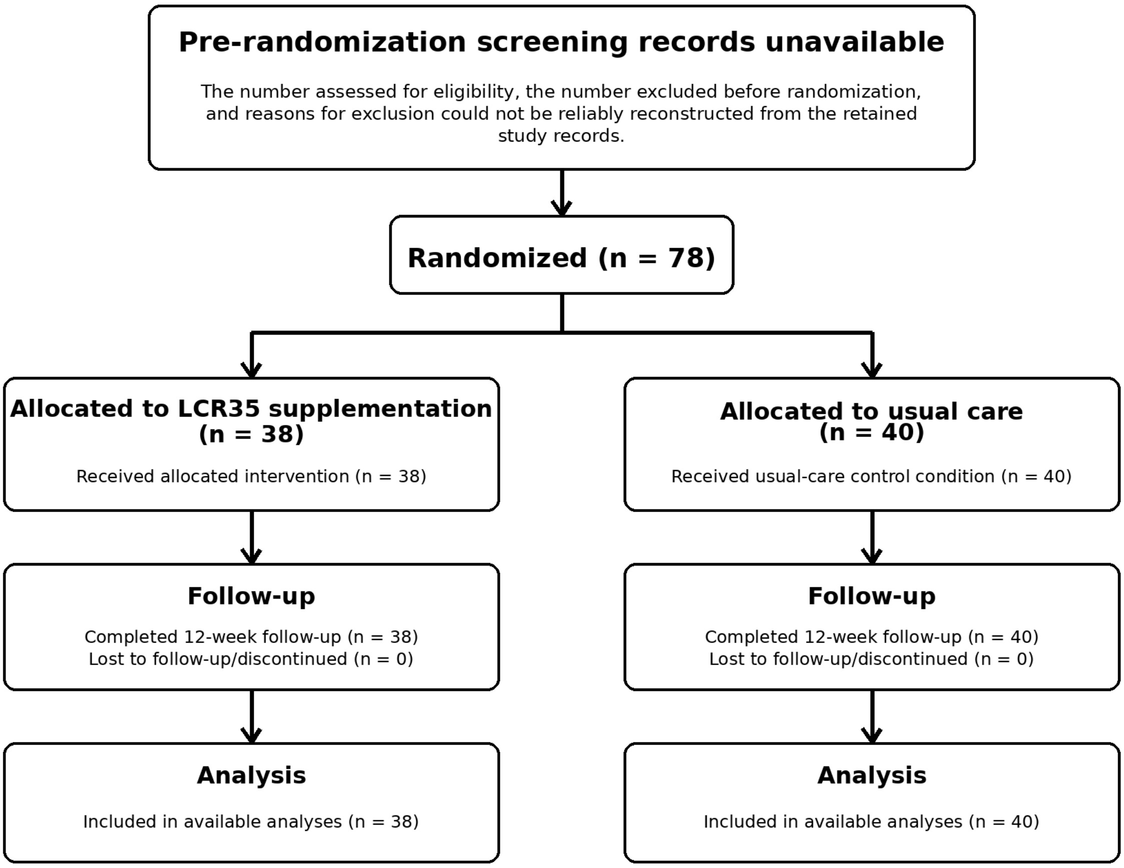

Supplementary Figure S1. CONSORT 2025 flow diagram showing participant allocation, follow-up, and analysis. Pre-randomization screening counts and reasons for exclusion were unavailable and could not be reliably reconstructed from the retained study records.
